# ^89^Zr-panitumumab PET imaging for preoperative assessment of ameloblastoma in a PDX model

**DOI:** 10.1038/s41598-022-23531-z

**Published:** 2022-11-10

**Authors:** Logan D. Stone, Adriana V. F. Massicano, Todd M. Stevens, Jason M. Warram, Anthony B. Morlandt, Suzanne E. Lapi, Hope M. Amm

**Affiliations:** 1 Department of Otolaryngology, University of Alabama at Birmingham, 1720 2nd Avenue South, VH G082, Birmingham, AL 35294 USA; 2 Department of Radiology, University of Alabama at Birmingham, 1824 6th Avenue South, WTI 310F, Birmingham, AL 35294 USA; 3 Department of Pathology, University of Alabama at Birmingham, 1802 6th Avenue South, NP 3548, Birmingham, AL 35294 USA; 4 Department of Oral and Maxillofacial Surgery, Section of Oral Oncology, University of Alabama at Birmingham, 1919 7th Avenue South, Birmingham, AL 35294 USA

**Keywords:** Biomarkers, Cancer imaging

## Abstract

Accurate assessment of tumor margins with specific, non-invasive imaging would result in the preservation of healthy tissue and improve long-term local tumor control, thereby reducing the risk of recurrence. Overexpression of epidermal growth factor receptor (EGFR) has been used in other cancers as an imaging biomarker to identify cancerous tissue. We hypothesize that expression of EGFR in ameloblastomas may be used to specifically visualize tumors. The aims of this study are to measure the specificity of radiolabeled ^89^Zr-panitumumab (an EGFR antibody) in vivo using patient-derived xenograft (PDX) models of ameloblastoma and positron emission tomography/computed tomography (PET/CT) scans. In PDX of ameloblastomas from four patients (AB-36, AB-37, AB-39 AB-53), the biodistribution of ^89^Zr-panitumumab was measured 120 h post-injection and was reported as the injected dose per gram of tissue (%ID/g; AB-36, 40%; AB-37, 62%; AB-39 18%; AB-53, 65%). The radiolabeled %ID/g was significantly greater in tumors of ^89^Zr-panitumumab-treated mice that did not receive unlabeled panitumumab as a blocking control for AB-36, AB-37, and AB-53. Radiolabeled anti-EGFR demonstrates specificity for ameloblastoma PDX tumor xenografts, we believe ^89^Zr-panitumumab is an attractive target for pre-surgical imaging of ameloblastomas. With this technology, we could more accurately assess tumor margins for the surgical removal of ameloblastomas.

## Introduction

Ameloblastomas (AB) are odontogenic tumors that display locally aggressive and destructive behavior. They are most likely to occur in the posterior mandible during the fourth or fifth decade of life^1–3^. While these tumors are benign, they can grow to large sizes and cause extensive damage to the bone and surrounding tissue. They are epithelial tumors believed to be derived from the epithelial rests formed during dental development. Conservative treatments have shown to have a recurrence rate of approximately 30%, due to incomplete resection of tumor^4–6^. There currently is no biomarker used or diagnostic strategies beyond biopsy. This makes surgical determination of accurate resection margins difficult, thus leading to the high recurrence rate^5,7^. New strategies for targeted imaging should be developed to improve accurate assessment of tumor margins. As a potential biomarker, the majority of ameloblastomas have been shown to be epidermal growth factor receptor (EGFR) positive^3,8–13^. One study that examined 193 cases of AB found that 85–100% of these cases were positive expression of EGFR^3^. This expression has been previously targeted in our lab^14^. Using EGFR as a biomarker for ameloblastoma would allow for the differentiation of tumor compared to normal tissue. Past experiments have used Food and Drug Administration approved antineoplastic antibodies that can be modified for use in fluorescence-guided surgery, including the anti-EGFR drugs cetuximab and panitumumab^14,15^. These FDA approved drugs are able to target the EGFR in cancer and have been approved for treatment in head and neck cancers and colorectal cancer using cetuximab and panitumumab, respectively^16^.

Here we use panitumumab radiolabeled with the radioisotope zirconium-89 (^89^Zr), which has been proven to be stable in vitro and in vivo^17^. This radioactive isotope allows for immuno-positron emission tomography^18^ imaging of the tumor, in particular as the longer half-life of ^89^Zr (compared to other radioactive isotopes) is compatible with the biodistribution and half-life of the antibody^17^. The use of ^89^Zr-panitumumab for PET-CT is a non-invasive method to monitor EGFR expression providing a useful way to identify EGFR-expressing tumors. Radiolabeled panitumumab has been studied for head and neck cancers and metastatic colon cancer as a means to determine lymph node involvement and tumor margins prior to an invasive surgery^3,17,19,20^, (NCT03764137). This strategy could provide better assessment of ameloblastoma tumor margins prior to surgery and reduce the invasive impact and outcomes for the patient.

## Results

### Preparation and characterization of ^89^Zr-panitumumab

**Figure 1:**
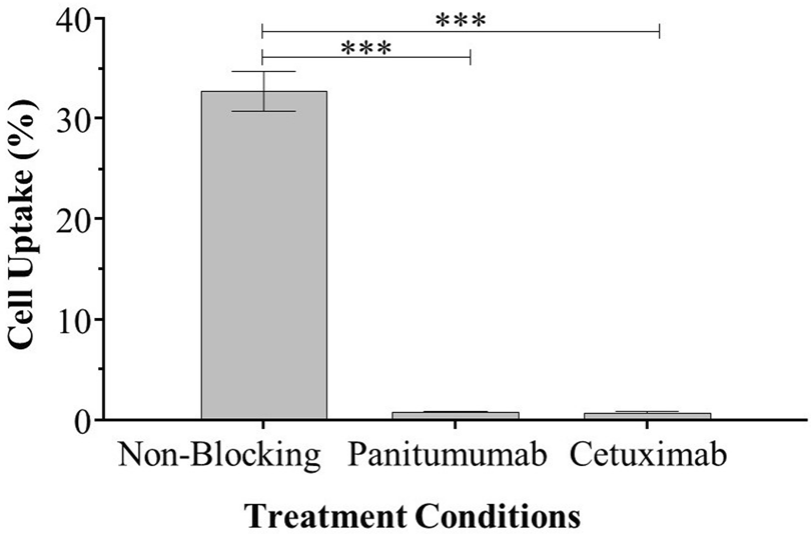
Cellular uptake of ^89^Zr-panitumumab in EGFR expressing SCC cells. Percent of administered activity detected in SCC-1-luc+ with and without blocking with unlabeled EGFR antibodies (***p < 0.0001).

Panitumumab was conjugated to 10 M fold of DFO-Bz-NCS and showed high radiochemical yield (> 95%) when labeled with ^89^Zr in all labeling conditions studied. Challenging the final product by adding DTPA into the solution did not affect the yield, confirming the high conjugation and labeling efficiency (results not shown). EGFR-positive SCC-1-luc+ cells were used to determine the ^89^Zr-panitumumab specific binding and immunoreactivity. Over 30% of the total ^89^Zr-panitumumab added was bound to the cells (32.75% ± 2.00%; Fig. 1), and this high binding was blocked when non-radiolabeled panitumumab was present (0.80% ± 0.03%, Fig. 1). The immunoreactivity ^89^Zr-panitumumab was over 85% (87.26% ± 15.98%) and thus panitumumab was not affected by the conjugation with 10 M fold of DFO-Bz-NCS or the radiolabeling process.

### Evaluation of EGFR expression using ^89^Zr-panitumumab and PET-CT images

Mice implanted with tumor samples from 4 separate ameloblastoma patients were injected with ^89^Zr-panitumumab with or without unlabeled panitumumab as a blocking control. During the experimental optimization a study was performed with biodistribution 5 days post-injection, however, the high background caused by the high blood pool compromised the imaging quality and consequent tumor visualization. The imaging quality was greatly improved by allowing the antibody to clear 2 more days from the body with imaging 7 days post-injection.

**Figure 2:**
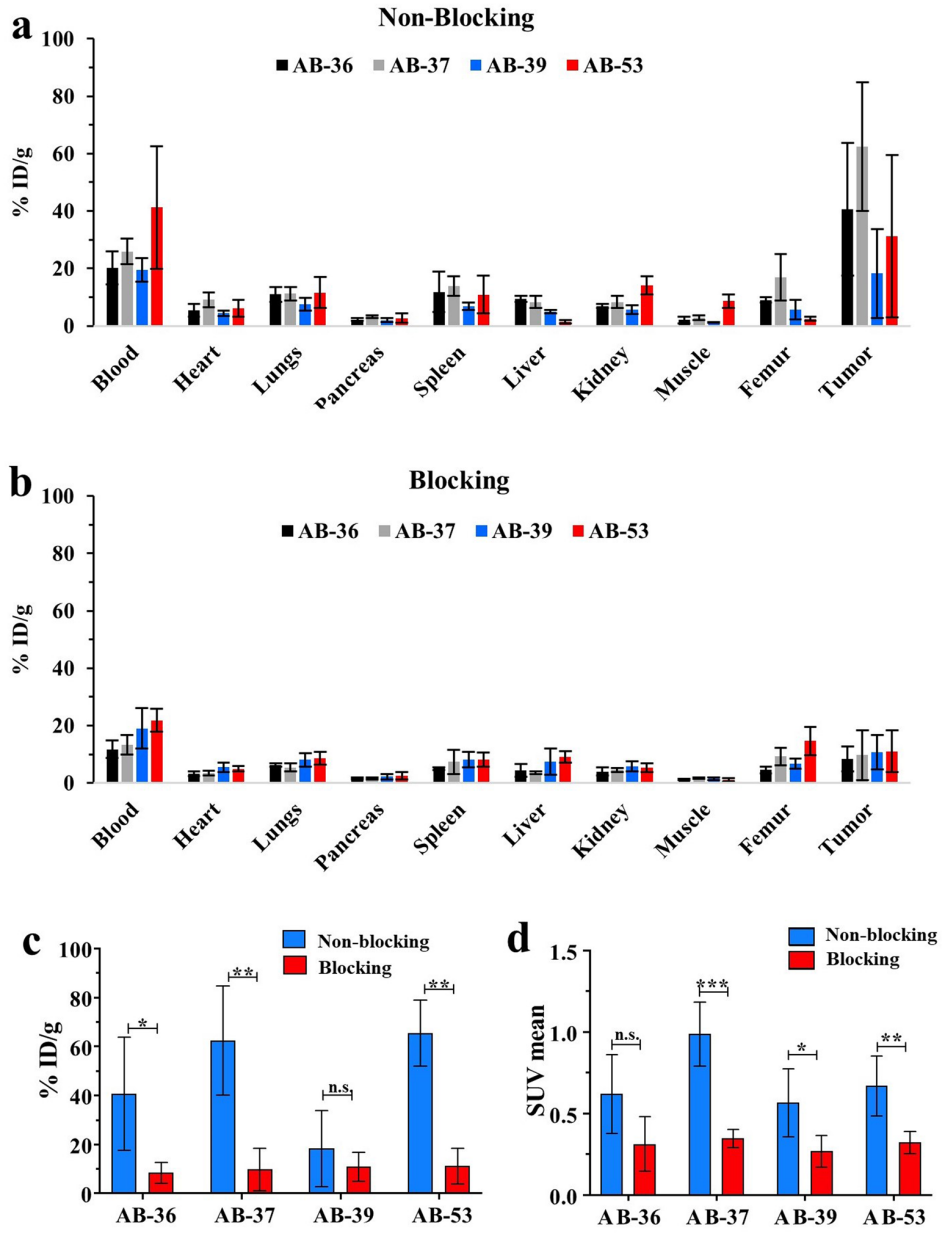
Detection of ^89^Zr-panitumumab in ameloblastoma patient derived xenograft models. Ameloblastoma tumor tissue from four patients was implanted in mice (AB-36, AB-37, AB-39, AB-53). All mice received ^89^Zr-panitumumab and were imaged at 7 days post injection. In the blocking group, 1 mg of unlabeled panitumumab was given 120 min prior to ^89^Zr-panitumumab. (**A**) Systemic biodistribution of ^89^Zr-panitumumab in non-blocking vs blocking groups. %ID/g = % injected dose per gram. (**B**) Average tumor uptake of ^89^Zr-panitumumab. (**C**) Standardized uptake value (SUV) in each tumor group. *p < 0.05; **p < 0.01; ***p < 0.001. Error bars indicate standard error.

**Figure 3:**
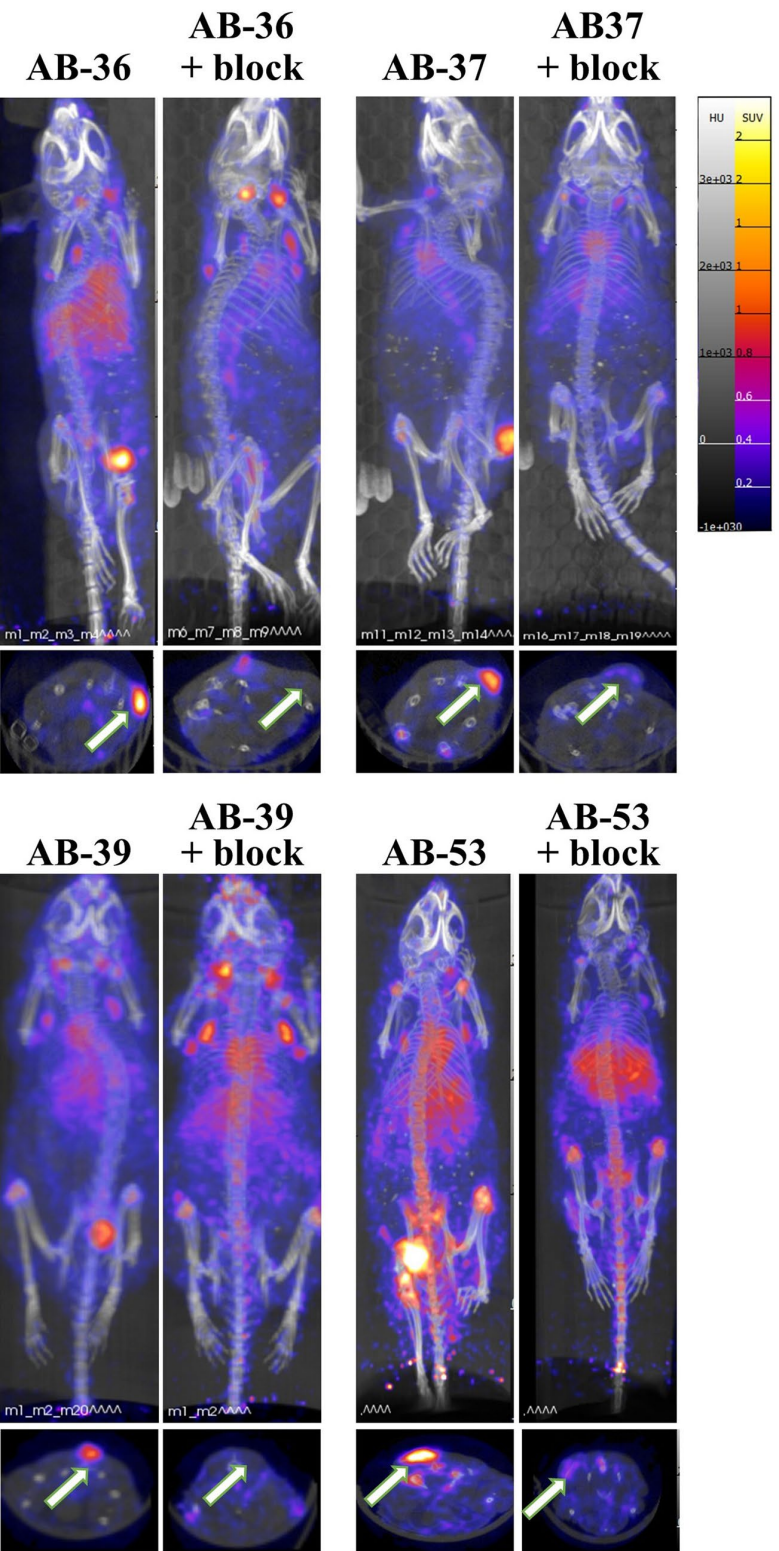
In vivo PET/CT imaging of ameloblastoma tumors. Representative MicroPET/CT images were obtained 7 days post injection. All animal received ^89^Zr-panitumumab. The blocking group (+ block) also received an unlabeled dose of panitumumab. Tumors were implanted in the flanks of mice. Maximum intensity is demonstrated in the upper panels, and the axial slice at the center of the tumor is shown in the bottom panels (as indicated by arrow). The scale, expressed as standardized uptake value (SUV), is at the far right.

Tumor uptake (%ID/g; percentage injected dose per gram of organ or tissue) in AB-36, AB-37 and AB-53 groups, but not the AB-39, was significantly higher in the non-blocking group compared to the blocking group, indicating the presence of EGFR expression in those tissues (Fig. 2A and B). However, the SUV mean (standard uptake value) for the AB-39 group was significantly higher for non-blocking group when compared to the blocking group (Fig. 2C). The SUV mean was also significantly higher in the non-blocking group for AB-37 and AB-53, corresponding with the significant tumor uptake (%ID/g). PET/CT images acquired showing ameloblastoma localization, as well as the difference between non-blocking and blocking groups, can be visualized in Fig. 3.

### Ex vivo histopathological confirmation of AB

**Figure 4:**
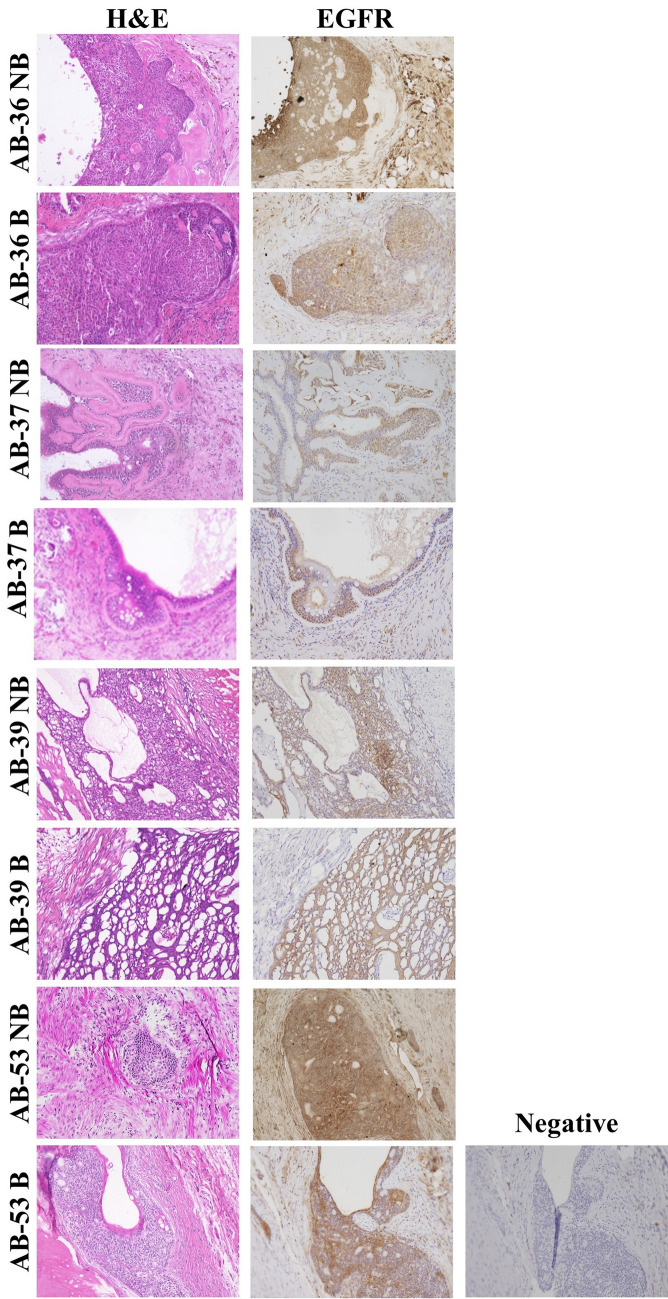
Ameloblastoma tumor tissue was confirmed in xenografts after resection by pathologic assessment (NB = non-blocking group; B = blocking group). Resected tumors, 7–9 weeks post implantation, were processed, embedded in paraffin and stained with hematoxylin and eosin (H&E, first column) or anti-EGFR for immunohistochemistry (second column) (20×). Negative controls (third column) show staining without primary antibody.

In order to confirm the presence of tumor in the PDX models, the implanted tissue was stained with hematoxylin and eosin (H&E). Microscopic examination demonstrated the presence of solid type ameloblastoma for each tumor specimen (Fig. 4). Immunohistochemistry also confirmed EGFR expression in the same areas confirmed by pathologist to be tumor (Fig. 4).

## Discussion

In the present study, we have confirmed that ^89^Zr-panitumumab has shown specificity for EGFR by binding to control EGFR-expressing cells, SCC-1-luc+ (Fig. 1)^14^. This binding can be blocked by the addition of unlabeled panitumumab. Previously, ^89^Zr-panitumumab, produced in the same manner, has shown specificity for EGFR expressed in A431 epidermoid carcinoma cells and HCT116 colon cancer cells in vitro and in vivo^17^. The specificity of ^89^Zr-panitumumab for EGFR has been demonstrated in multiple EGFR+ cell lines and allows us to target EGFR in ameloblastomas^17,21,22^. Our group has previously shown that ameloblastoma cells expresses EGFR at a similar level to SCC cells^14^.

We previously targeted EGFR in ameloblastomas using fluorescently labeled cetuximab, another EGFR antibody, which showed specificity for ameloblastoma in vitro and in vivo^14^. While targeted fluorescence imaging is attractive due to being non-invasive and having low toxicity, it is unknown if near infrared fluorescence imaging is feasible within bone^23^. Ameloblastoma occurs within in the bones of the jaw, primarily in the mandible^1,2^. Imaging tumors within bone remains challenging with no consensus regarding the optimal imaging strategies. In head and neck squamous cell carcinoma (HNSCC) patients with mandibular tumor involvement, CT, magnetic resonance imaging (MRI), ^18^F-fluorodeoxyglucose (FDG)-PET, SPECT/CT, and PET imaging have all been evaluated, and each approach has advantages and disadvantages^24,25^. Radiolabeled EGFR antibodies have shown promise in imaging tumors where fluorescent imaging may not be feasible. Zirconium-89 is a positron emitter with a relatively long half-life (3.3 days)^17^. Previous pharmacokinetic studies have demonstrated that it takes 5–7 days to obtain optimal tumor-to-non-tumor ratios for antibody localization in vivo, making zirconium-89 compatible with panitumumab as a PET agent^17,26^.Small animal PET with radiolabeled antibodies, such as with ^89^Zr-panitumumab, provide the benefit of specificity for tumor tissue. When combined with the high sensitivity of PET, this technique allows for imaging of the tumor area and margins. The current imaging modalities used for ameloblastoma include radiography, CT, and MRI^27,28^. In some cases, contrast-enhanced MRI, cone beam CT (CBCT), or FDG-PET may be used^29–33^. However, the former modalities lack specificity for tumor tissue, and solid ameloblastomas only demonstrate mild or moderate FDG uptake. This is likely due to the low proliferative state and glucose use of these tumors. The ability to specifically and visibly label tumor cells with precision, regardless of their proliferative state, would be beneficial for patients facing extensive surgeries. ^89^Zr-panitumumab provides specificity to EGFR-expressing tumors, like ameloblastomas, and the ability to visualize tumors prior to surgery.

In the current study, we show ^89^Zr-panitumumab localized to ameloblastoma tumors using PET/CT scans (Fig. 2). This demonstrates ^89^Zr-panitumumab can bind to ameloblastoma PDX tissue in vivo similar to localization shown in previous models with cetuximab-IRDye800. Biodistribution and SUV data showed significant localization to in vivo ameloblastoma tissue compared to other organs and sites within the mouse (Fig. 2). Differences in ^89^Zr-panitumumab uptake occurred in the tumor tissue between the blocking and non-blocking groups demonstrating the specificity for EGFR. The PET images demonstrate that ^89^Zr-panitumumab can be used to identify the tumor tissue from four distinct patients with ameloblastoma in vivo using the mouse PDX model (Fig. 3). Ex vivo the presence of ameloblastoma tumor and expression of EGFR was confirmed in each AB PDX (Fig. 4). These findings establish that ^89^Zr-panitumumab is tumor-specific and highly sensitive to detecting ameloblastoma tumors in vitro.

Currently it is difficult for surgeons to evaluate these ameloblastoma tumors radiographically. In general, 30% of head and neck tumor resections were found to have positive margins during pathology review^5^. Ameloblastoma margins are often assessed by qualitative visual inspection or based on radiographs of the resected specimen^6^. Frozen section histology has also been used to evaluate the soft tissue adjacent to intrabony tumors to assess margin status^34^; however, the assessment of bony resection margins requires decalcification and sectioning, which requires at least one day precluding frozen section analysis on bone margins. Margins of 1–2 cm are currently considered adequate for curative resection^35^. However, a 1 cm margin beyond the radiographically-visible disease has been associated with a 29% recurrence rate^32^. Because of this, over-aggressive resection with wide margins up to 3 cm for ameloblastoma has been advocated to avoid recurrence. However, the over-resection of healthy tissues results in unnecessary facial deformity, malocclusion, neurosensory disturbances, and speech and swallowing dysfunction. In many cases, radical resection requires extensive reconstruction, including microvascular composite free tissue transfer^8^. The management of recurrences following reconstruction may require the sacrifice of a bone graft or soft tissue constructs. This leads to additional reconstructive procedures and donor site morbidity. The ability to accurately assess tumor margins using targeted imaging could help to preserve healthy tissue and improve long-term local tumor control. Here we provide proof-of principle data that ameloblastoma tumors can be specifically visualized in vivo and ^89^Zr-panitumumab may provide precise preoperative imaging of tumor margins.

The principal limitations of this study include limited sample size and subcutaneous implantation of the primary tumor xenografts. Although the number of ameloblastoma patients recruited to this study was limited, we were able to detect significant localization of ^89^Zr-panitumumab to ameloblastoma tumors in vivo, which would aid surgeons in planning successful resections. For this preclinical study, the ameloblastoma xenografts were implanted in the flanks of mice. Although subcutaneous xenografts, including the PDX models used here, provide a useful model of the disease and proof-of-principle data, these models do not replicate the tumor environment. Ameloblastomas occur within an intraosseous environment, primarily the mandible. Studies in HNSCC have shown the benefits of using PET/CT in visualizing the bone involvement and tumor extension into bone^36–38^. Studies used ^18^F-FDG or ^18^F-NaF PET/CT to accurately access the mandibular extension of HNSCC. This approach allows for the benefits of tumor labeling provided by PET imaging and higher anatomical resolution provide by CT^39,40^. However, these PET modalities lack specificity as they are based on the metabolic capacity of the tissue or bone. Our approach utilizes an immuno-PET agent, ^89^Zr-panitumumab, which binds to EGFR expressed by the ameloblastoma tissue^14,17^. The first human clinical trials using ^89^Zr-panitumumab for imaging showed that it has clinical application to cancers that overexpress EGFR and can be safely used in humans^19^. Other papers have demonstrated that ^89^Zr labeled antibodies have a wide range of clinical applications that need to be further explored to efficiently use it in a clinical setting^41^. Our study demonstrates that ^89^Zr-panitumumab is taken up by AB xenografts. This suggests that further studies should be done to examine the efficiency in the mandible of humans.

## Conclusion

In conclusion, our data have shown that radiolabeled anti-EGFR antibody, ^89^Zr-panitumumab, can be used to effectively identify ameloblastoma tumor tissue in vivo. This was demonstrated through significant localization of ^89^Zr-panitumumab to ameloblastoma PDX tissue as measured by biodistribution and SUV. This approach takes advantage of the EGFR expression seen in ameloblastoma tumors, while providing feasibility of utilizing advanced imaging for ameloblastoma detection. We believe the technologies we have described, once translated into the clinical setting, will allow surgeons to more confidently excise ameloblastomas by accurately assessing the tumor location and preserving normal tissue, thereby improving long-term local tumor control and, ultimately, patient outcomes.

## Materials and methods

### Tumor specimens and preparation

Tissue samples were collected from consented patients with a diagnosis of ameloblastoma, who underwent surgical resection at the University of Alabama at Birmingham (UAB) between 2018 and 2020. During the study period, patients were identified with a diagnosis of primary or recurrent ameloblastoma requiring a microvascular free flap. Patients included in this study had solid ameloblastoma with mandibular bone involvement with negative lymph nodes.

The UAB Institutional Review Board independently reviewed and approved the present study, and all patients provided written informed consent. The ablative surgeon (A.B.M.) obtained the tumor tissue for the study distant from the specimen edges using a method that did not interfere with the pathologist’s ability to assess the surgical margins. To confirm the presence of the tumor before implantation, a portion of the tissue was analyzed by a board-certified pathologist (T.M.S.) using standard frozen section techniques and the World Health Organization diagnostic criteria for AB^42^. All patients enrolled in the study had a solid/multicystic ameloblastoma of the mandible. AB-37, AB-39, and AB-53 were the follicular histopathological subtype; AB-36 was a desmoplastic subtype. Once the tumor presence had been confirmed, the explants were dissected into 2 × 2-mm pieces, rinsed in Dulbecco’s modified Eagle medium (DMEM)/F-12 supplemented with penicillin/streptomycin and amphotericin (ThermoFisher, Waltham, MA). The remainder of the surgical specimens were processed by the pathology department in accordance with the standard of care for surgical pathologic specimens^14^.

### Antibodies and reagents

The production of [^89^Zr]oxalate was carried out the University of Alabama at Birmingham, Cyclotron Facility using Y sputtered solid targets as described previously^43^. Panitumumab (Vectibix®) was purchased from Amgen (Thousand Oaks, CA). Desferrioxamine-p-benzyl-isothiocyanate (DFO-Bz-NCS) was purchased from Macrocyclics (Dallas, TX). Dimethyl sulfoxide (DMSO) and sodium carbonate were purchased from Sigma-Aldrich (St. Louis, MO). HEPES was purchased from ACROS Organic (Fair Lawn, NJ). All other chemicals were purchased from Fisher Scientific (Hampton, NH) unless stated otherwise.

### Cell culture

Squamous cell carcinoma-1 1uciferase-positive (SCC-1-luc+) EGFR-positive cells were kindly donated by Dr. Jason Warram, PhD and cultivated in Dulbecco’s modified Eagle’s medium containing 10% fetal bovine serum (FBS) and supplemented with 50 µg/mL gentamycin in a humidified incubator with 5% CO_2_ at 37 °C^44^. All other reagents for cell culture were purchased from Fisher Scientific (Hampton, NH).

### Preparation of ^89^Zr-panitumumab

Conjugation of DFO-Bz-NCS to panitumumab and radiolabeling with [^89^Zr]oxalate were performed following previous methods^26,45^ Briefly, tenfold molar excess of DFO-Bz-NCS dissolved in DMSO was incubated with panitumumab in 0.1 M sodium carbonate buffer (pH 9) at 37 °C for 1 h. After conjugation, the DFO was removed by Zeba spin desalting columns using 1 M HEPES buffer pH 7.1–7.3 via Zeba spin desalting columns (40 kDa Molecular Weight Cut-Off, Thermo Scientific, Rockford, IL). Protein concentration was quantified using a bicinchoninic acid (BCA) assay (Thermo Scientific, Rockford, IL). The purified DFO-panitumumab was labeled with neutralized ^89^Zr-oxalate using desalting columns under the following conditions: 12.5 μg of mAb and 50 μCi of ^89^Zr (12.5 μg/50 μCi); 6.25 μg/50 μCi; 5 μg/50 μCi and 2.5 μg/50 μCi (0.148, 0.296, 0.370 and 0.740 MBq per µg mAb respectively) in a final volume of 50 µL at 37 °C for 1 h.

The radiochemical yield was determined by instant thin-layer chromatography (iTLC) using 50 mM DTPA as the mobile phase. The labeled antibody with radiochemical yields^46^ ≥ 95% chromatography was used for in vitro and in vivo studies without further purification. When needed (RCY < 95%), the labeled antibody was purified using Zeba spin desalting columns.

### Immunoreactivity and specific cell binding in vitro

The specific binding of ^89^Zr-panitumumab was assessed in SCC-1-luc+ cells in vitro. The immunoreactivity and specificity was previously shown by Chang et al.^17^. Cells (2.5 × 10^4^) were seeded in a 12-well plate and incubated in a humidified incubator with 5% CO_2_ at 37˚C for 48 h. Half of the wells received 25 ng of ^89^Zr-panitumumab in 1 mL of culture medium (total binding) and half received the same solution plus 25,000 ng of non-radiolabeled panitumumab in 1 mL of culture medium (competitive/blocking binding). The cells were incubated for 1.5 h at 37 °C; 5% CO_2_. At the end of incubation, the cells were washed twice with cold PBS, trypsinized, and assayed in a gamma counter. The percentage of ^89^Zr-panitumumab bound to the cells in presence or absence of competitor was calculated.

### Animal model PDX

Athymic mice (Envigo, East Millstone, NJ) aged 3–6 weeks, were obtained and housed in accordance with the UAB Animal Resource Program guidelines, after approval by the Institutional Animal Care and Use Committee. The mice were anesthetized with isoflurane and incision sites were prepared with 3M Durprep, Iodine Povacrylex (Fisher Scientific) and ethanol. Prior to surgery, the mice were treated with carprofen. An incision was made between the skin and muscle of the left flank, followed by developing a subcutaneous pocket at this site^14^. A tumor piece (2 × 2 mm) was placed in the pocket and the wound was closed with staples (Fine Science Tools, Foster City, CA). The tumors were allowed to establish for 4–6 weeks after PDX implantation. Number of mice per group varied based on patient tissue availability (n = 5 in all groups except AB-39 blocking where n = 4).

### Evaluation of EGFR expression using PET/CT images

For each PDX model, mice were divided into 2 groups of 5 mice (except where noted). The non-blocking group received 3.7 MBq (100 µCi; 5 μg of antibody) of ^89^Zr-panitumumab via tail vein, and the blocking group received 1 mg of unlabeled panitumumab 120 min prior to receiving the same radioactive dose of ^89^Zr-panitumumab. Static PET images were acquired 7 days post injection for 20 min using GNEXT PET/CT (SOFIE, Dulles, VA). The images were reconstructed using a MLEM 3D protocol and the standard uptake value (SUV) of each tumor was calculated using VivoQuant software (Invicro, Boston, MA). After image acquisition, mice were euthanized, the main organs were harvested, weighed, and counted in a gamma counter to calculate the percentage of injected dose in each organ. All the tumors were paraffin embedded and used for pathologic analysis.

### Immunohistochemistry

Formalin-fixed, paraffin embedded (FFPE) tumors were cut (5 mm sections), deparaffinized, and rehydrated. The sections were placed in citrate antigen retrieval buffer (R&D Systems, Minneapolis, MN) for 20 min at 97 °C and blocked in 3% hydrogen peroxide for 10 min. The samples were incubated with EGFR antibody (A10, Santa Cruz, 1:50) overnight at 4 °C. Horseradish peroxidase polymer-conjugated antibody was applied for 10 min and detected using diaminobenzidine tetrachloride with hematoxylin counterstain (Invitrogen, Waltham, MA). The negative control was processed with secondary antibody and without primary antibody.

### Statistical analysis

Statistical analysis was performed using Student’s t-test (p < 0.05) for single comparison All analyses were performed on GraphPad Prism V9 (GraphPad Software Inc, La Jolla, CA).
